# Spinal metastasis in head and neck cancer

**DOI:** 10.1186/1758-3284-4-36

**Published:** 2012-06-20

**Authors:** Gregory M Trilling, Hyongyu Cho, Mohamed A Ugas, Samerah Saeed, Asia Katunda, Waseem Jerjes, Peter Giannoudis

**Affiliations:** 1 Barts and The London School of Medicine and Dentistry, Queen Mary, University of London, London, UK; 2 Department of Surgery, Al-Yarmouk University College, Baghdad, Iraq; 3 Department of Surgery, UCL Medical School, London, UK; 4 Leeds Institute of Molecular Medicine, University of Leeds, London, UK; 5 Academic Department of Trauma and Orthopaedic Surgery, Leeds Teaching Hospitals NHS Trust, Leeds, UK

## Abstract

**Background:**

The incidence of head and neck cancer is relatively low in developed countries and highest in South East Asia. Notwithstanding advances in surgery and radiotherapy over the past several decades, the 5-year survival rate for head and neck cancer has stagnated and remains at 50–55%. This is due, in large part, to both regional and distant disease spread, including spinal metastasis. Spinal metastasis from head and neck cancer is rare, has a poor prognosis and can significantly impede end-stage quality of life; normally only palliative care is given.

This study aims to conduct a systematic review of the evidence available on management of spinal metastasis from head and neck cancer and to use such evidence to draw up guiding principles in the management of the distant spread.

**Methods:**

Systematic review of the electronic literature was conducted regarding the management of spinal metastasis of head and neck malignancies.

**Results:**

Due to the exceptional rarity of head and neck cancers metastasizing to the spine, there is a paucity of good randomized controlled trials into the management of spinal metastasis. This review produced only 12 case studies/reports and 2 small retrospective cohort studies that lacked appropriate controls.

**Conclusion:**

Management should aim to improve end-stage quality of life and maintain neurological function. This review has found that radiotherapy +/− medical adjuvant is considered the principle treatment of spinal metastasis of head and neck cancers.

There is an absence of a definitive treatment protocol for head and neck cancer spinal metastasis. Our failure to find and cite high-quality scientific evidence only serves to stress the need for good quality research in this area.

## Introduction

The incidence of head and neck cancer is relatively low in developed countries and highest in South East Asia. There are marked regional variations in the incidence of head and neck cancers, with rates ranging from 8 per 100,000 in the Thames and Oxford regions to 13–15 per 100,000 in Wales and in the North Western region [1-4]. It is rare before the age of 45 [1-3]. Risk factors for head and neck cancer include tobacco, alcohol, betel quid, and Human Papilloma Virus (HPV) [1-3]. Head and neck cancers affecting the oropharynx, tonsil, and base of the tongue are on the increase in the young. This is thought to be due to changes in sexual behaviour leading to increased HPV transmission [1].

There is no consensus over which cancers to categorise as head and neck cancer. This study includes the ICD 10 codes; C00-C010 and C12-C14. These codes account for the following sites: oral cavity, oropharynx, laryngopharynx, hypopharynx and salivary glands in addition to other and ill-defined sites involving the lip, oral cavity and pharynx. Cancers involving the rest of the head and neck, including nasopharynx, cranial sinuses, thyroid, brain and eyes were out of the scope of this study.

Squamous cell carcinomas (SCC) constitute 95% of cancers in this area [1]. The most common sites for SCC are the tongue (oral or base) followed by the floor of the mouth, retromolar area (trigone), tonsils and lower lip [1]. SCC cancer is most likely known to metastasize and recur and is associated with significant morbidity and mortality [1].

Cancers of the salivary glands demonstrate a different cytological profile. Types include carcinoma ex pleomorphic adenoma, adenoid cystic carcinoma (ACC) and acinic cell carcinoma. Pleomorphic adenoma (PA, also known as a benign mixed tumour) is the commonest benign tumour of the salivary glands [1]. They are characterized by indolent growth and are painless. Carcinomas ex pleomorphic adenoma (carcinosarcoma and carcinoma ex-mixed tumour) however, are a rare and aggressive tumour with a reported 5-year survival rate of 50% and haematogenous spread [1]. ACC or adenoid cystic carcinoma (also known as a cylindroma) is a rare and malignant tumour that occurs mainly in the non-parotid salivary glands [1,2]. Acinic cell carcinoma is a rare neoplasm which mainly affects the salivary glands (in particular the parotid gland); it constitutes to 1-3% of all the salivary gland tumours [1]. This cancer is normally found to be low grade [1-3] and painless with good prognosis [1-3].

Head and neck cancer carries significant morbidity, affecting appearance and function (i.e. swallowing, speaking and breathing) and consequently patients may experience depression and poor nutrition [1-29]. Surgery is the most definitive method used to treat patients with this unforgiving disease. Furthermore, this disease can be managed using adjuvant therapies such as chemotherapy (CT), radiotherapy (RT), chemoradiotherapy (CR) or photodynamic therapy (PDT). Factors to be taken in consideration for surgery are tumour type, staging, site, likelihood of metastasis, patient age, medical status, ability to tolerate treatment and lifestyle (i.e. smoking and alcohol). RT is often given postoperatively to provide better control of the surgical margins [8]. Chemotherapy is usually offered as a palliative treatment however this situation has changed with introduction of cis-platinum, and now there is potential for it to be used as treatment [8]. Photodynamic therapy is now the forth modality (after surgery, RT and CT) when managing this disease. Other therapies in development include immunotherapy and gene therapy [8].

Mortality in most countries for cancer of the oral cavity and oropharynx is around 50-55% within 5 years. Prognosis is worse in older patients and for difficult to access cancers (i.e. laryngopharynx and hypopharynx), both carry a higher mortality [10,17]. Cancers affecting the lip have the highest 5-year survival rate (90%) most likely due to early detection and accessibility, whereas hypopharyngeal tumours have the lowest survival rates [17]. Females tend to have a better 5-year survival rate for cancers of the oral cavity and oropharynx, than males. Patients who present with advanced TNM staging also have a worse prognosis [17]. Additionally, extra capsular spread have worse prognosis [9].

Metastasis from a primary head and neck cancer occur less often in comparison to other cancers due to low incidence of haematogenous spread [1]. SCC are the most likely tumour to spread and the most common site of metastasis for head and neck cancer is the lungs [1,12,19]. There are many tumours that are known to commonly spread to the spine [13] and overall the incidence of spinal metastasis is approximately 5% [1,13]. Spread from other malignancies to the spine is reported to be higher, especially from breast (20%), prostate, lungs (12%), kidney and primary thyroid cancers [20,22]. However, metastasis arising from a primary head and neck cancer to the spine is not widely reported [13].

### Spinal metastasis

Bone, especially the spine [1], is the third commonest site of metastasis after the lungs and liver [1]. Incidence of spinal metastatic neoplasm outnumbers primary spinal neoplasms by more than twenty-to-one [1]. The vast majority originate from breast, lung, prostate or primary renal tumours [1,2]. Prevalence of spinal metastasis is highest among individuals between the 4^th^ and 7^th^ decade of life [1,2,23]. Males are more likely to be afflicted than females; this is thought to be reflective of the higher prevalence of lung cancer in males and the higher prevalence of prostate cancer relative to breast cancer [9-45].

Spinal metastasis (SM) typically affect the thoracic (60-80%), lumbar (15-30%) and cervical spine (<10%) with the preferred route of metastasis to the spine being via the arterial or venous -Batson’s venous plexus - vessels often resulting in multifocal lesions [1,29]. Direct infiltration from paraspinous disease or, less commonly, through the cerebrospinal fluid [24,28] are also potential routes of metastasis. The vertebral body (85%) is the commonest site for initial spinal metastasis involvement; the posterior aspect of which is preferentially involved (66%). The paravertebral spaces (10-15%) and the epidural space (<5%) are also initial sites of metastatic involvement [24,28,29].

Vertebral metastasis are asymptomatic and may be incidental findings following routine bone scans in patients presenting with systemic disease [21,28]. Classical clinical symptoms develop with the progression of spinal metastatic disease and are consequences of metastatic infiltration and/or compression of paravetebral, osseous and neural tissue [21].

Spinal canal to spinal cord ratio is smallest in the thoracic spine hence SCCs are more common in the thoracic spine [21]. The most frequent cause of SCC and nerve root compression is the expulsion of metastatic tissue and/or detritus of bone into the spinal canal or neural foramina following metastatic infiltration and ensuing partial collapse of the vertebral body. On infrequent occasion, the metastatic tissue may break into the spinal canal and cause SCC without assaulting the vertebral body’s structural integrity [21].

The chief presenting symptom of spinal metastasis is pain (83-95%) [24,28]. Spinal metastasis typically presents with progressive, unremitting pain of gradual onset, worse at night and improving with activity and anti-inflammatory medication. Tenderness of the spine in the affected area is common. Pain can also be non-mechanical, radicular or neuropathic especially in the case of intradural metastasis [21,24,28].

Neurological dysfunction due to anterior displacement of the spinal cord is also common. A typical early complaint of limb heaviness is confirmed by weakness in 1 ≥ muscle groups on physical examination [24]. Posterior displacement of the spinal cord and impingement against the lamina results in sensory dysfunction [21,28] is commonly an advanced feature in the clinical course of spinal metastatic disease and can be accompanied by profound motor dysfunctions such as paralysis, anal and urethral sphincter dysfunction and sexual malfunction [24,28].

Plain X-ray is used to identify metastatic lesions, tumour masses and evaluate spinal stability [25,28]. X-rays are insensitive in early spinal metastatic diagnosis as 30-50% demineralisation of bone is required before lytic lesions become apparent on radiographic film [24,25,28]**.** Magnetic resonance imaging (MRI) is the gold-standard imaging for diagnosis of spinal metastasis. It renders exquisitely detailed multiplanar imaging allowing the visualisation of metastatic infiltration and/or compression of paravetebral, osseous and neural tissue [24,25,28]. T1- and T2-weighted imaging as well as contrast-enhanced and fat-suppressed studies in all three planes should aid diagnosis [28].

Computed tomography (CT) imaging is an excellent modality in assessing the osseous spine and has a high degree of accuracy (90% sensitivity, 100% specificity), [24] in identifying metastatic lesions, vertebral destruction and spinal stability. CT angiography is ideal in identifying spinal metastasis from highly vascular primary malignancies [28]. Bone scintigraphy is also used to screen for bone metastasis. However, despite its documented 62-89% sensitivity, it should be noted that bone scintigraphy measures abnormalities in bone metabolism and does not, therefore, possess a high specificity in identifying spinal metastasis [24]. MRI and/or CT should be used to authenticate suspected spinal metastasis. Single-photon emission computed tomography (SPECT) and fluorodeoxyglucose positron emission tomography (FDG-PET) are both superior to bone scintigraphy and are used in surveillance of patients suspected of SM [24]. Finally biopsy under CT fluoroscopic guidance is crucial in staging SM and formulating surgical/medical treatment plan.

This study aims to conduct a systematic review of the evidence available on management of spinal metastasis from head and neck cancer, excluding cancers of the nasopharynx, cranial sinuses, thyroid, brain and eyes.

## Materials and methods

A systematic literature research was conducted using electronic databases such as PubMed, Google Scholar and Science Direct. English as well as non-English studies were retrieved using the following MeSH and non-MeSH terms:

· Oral, Mouth, Tongue, Lingual, Sublingual, Lip, Labial, Salivary Gland/Ducts, Parotid Gland, Mandibular, Submandibular, Piriform Sinus, Oropharynx, Larynx, Hypopharynx, Bone, Head and Neck Cancer/Carcinoma/Neoplasm/Pathology, Mucoepidermoid, Squamous Cell Carcinoma/Primary/Secondary/Diagnosis, Adenocarcinoma/Secondary, Adenoma, Adenoid Cystic Carcinoma, Pleomorphic/Pathology, Acinic Cell Carcinoma, Radiotherapy (RT), Steroids, Chemotherapy (CT), Palliative Care

· Spinal, Vertebral/Thoracic/Lumbar/Cervical, Lymphatic Metastasis/Pathology, Spinal Cord Compression/Aetiology/Therapy

· Male/Female, Adult, Retrospective Studies, Case Studies/Reports, Follow-Up Studies

The inclusion criteria were as follows: original research studies and case studies/reports discussing interventions for distant metastasis in head and neck cancer – medical, surgical, radiological; operative care, treatment outcomes and prognosis.

In order to ensure that no relevant publications were missed; references of articles generated by our primary searches were scanned and reviewed for potential inclusion in this review. The initial searches yielded 93 articles; we excluded studies that only addressed treatment of the primary malignancy, metastasis other than spinal ones. Furthermore, some of the cancers in head and neck area were also excluded; this includes nasopharynx, thyroid, cranial sinuses, brain and eyes cancers. We also excluded studies not available in full text or in the English language.

The data were gathered from the selected articles: primary site, grade, stage of primary tumour, intervention for the primary, location of spinal metastasis, timeframe to metastasis, interventions for the metastasis and outcome following treatment (Table1, 2, 3, 4 and 5).

**Table 1:** Summary of the study location, study types and cancer types

**Author, year**	**Study location**	**Study type**	**No. patients**	**Cancer types**
Thomas, 1965 [30]	USA	Case Report	2	Carcinoma ex pleomorphic adenoma
Riela, 1983 [15]	USA	Case Report	1	ACC
Ampil, 1994 [31]	USA	Retrospective Cohort Study	4	SCC
Preciado, 2002 [20]	USA	Retrospective Cohort Study	6	SCC, ACC
Birkeland, 2003 [15]	Denmark	Case Report	1	ACC
Mendes, 2004 [18]	UK	Case Report	3	SCC
Manoj-Thomas, 2006 [13]	UK	Case Report	1	Carcinoma ex pleomorphic adenoma
Lee, 2007 [32]	New Zealand	Case Report	2	SCC
Vahtsevanos, 2007 [33]	Greece	Case Report	3	SCC
Vidyadhara, 2007 [34]	India	Case Report	1	Acinic cell carcinoma
Ye, 2007 [12]	South Korea	Case Report	1	Carcinoma ex pleomorphic adenoma
Törnwall, 2008 [19]	Finland	Case Report	1	SCC
Yu, 2008 [35]	China	Case Report	1	SCC
Le Manarc’h, 2009	France	Case Report	1	Acinic cell carcinoma

**Table 2:** Patients with squamous cell carcinoma as primary head and neck cancer

**Author, year**	**Patient No.**	**Patient age**	**Primary site, grade**	**Primary stage**	**Intervention for primary site**	**Location of spinal metastasis**	**Time frame of spinal metastasis**	**Signs + symptoms of spinal metastasis**	**Intervention for spinal metastasis**	**Outcome**	**Other metastasis**	**Mortality**	**Cause of death**
Ampil, 1994 [31]	1	60	Hypopharynx SCC	T2 N2	None	T6	0 mo	Back pain, lower limb motor deficit	Laminectomy, adjuvant RT	Significant response	-	4 mo	-
	2	56	Oropharynx SCC	T3 N3	Pre-op RT	L3-L4	-	Back pain, lower limb motor deficit	RT	No response, alive at 5 mo	-	-	-
	3	40	Oropharynx SCC	T4 N3	RT	C3-C4	0 mo	-	RT	No response	-	1 mo	-
	4	66	Larynx SCC	-	Laryngectomy	T9	-	Back pain, lower limb motor deficit	RT	Complete response, alive at 88 mo	-	-	-
Preciado, 2002 [20]	5	50	Tonsil SCC	T3 N2b	RT (70 Gy), CT (cisplatin, 5FU)	C6, C7, T1	6 mo	Grade I: weakness, pain	Decompression, fusion, steroids	Grade 0; full use of arm	Temporal bone, lung	13 mo	Disseminated disease
	6	55	Base of tongue SCC	T4 N2	-	C5-C6	11 mo	Grade I: weakness, pain	Steroids[Refused RT, surgery]	Grade III; non-ambulatory, pain	None	3 mo	-
	7	55	Base of tongue SCC	T4 N2c	-	C5, T2	37 mo	Grade I: weakness, pain	RT (20 Gy) with Strontium chloride Sr-89	Grade 0; improved pain	Lung	1 mo	-
	8	61	Parotid SCC	-	-	T11-L5	11 mo	Low back pain	RT (2.5 Gy), steroids	Mildly improved pain	None	2 wk	-
	9	56	Hypopharynx SCC	T4 N2c	-	T12-L1	38 mo	Low back pain	RT (20 Gy), IV dexamethasone	Improved pain	None	5 mo	-
Mendes, 2004 [18]	10	73	Tongue SCC	T2 N0	Wide excision	C4	4 mo	Neck pain, left arm weakness	RT (20 Gy), CT (cisplatin, 5FU)	Improved pain, continued weakness	Regional	4 mo	-
	11	63	Vocal cord SCC	T1a N0	RT (50 Gy)	C5	14 mo	Shoulder pain, neck tenderness, weakness	RT (20 Gy)	Moderately improved pain	Regional	6 wk	-
	12	53	Pyriform fossa SCC	T3 N2c	CT (cisplatin, 5FU), CR (65 Gy with cisplatin)	C2, C4, T1-T4	5 mo	Cervical/ thoracic back pain	RT (20 Gy)	Pain and neurological symptoms initially improved	Skull base, cerebellopontine cistern, right temporal lobe	4 wk	-
Lee, 2007 [32]	13	52	Tongue SCC	-	RT (60 Gy), CT (cisplatin)	T10	11 wk	Numbness, weakness	Steroid, RT	Persisted neuro deficits	Regional	1 mo	Disseminated disease
	14	60	Tongue SCC	-	Subtotal glossectomy, bilateral neck dissection, reconstruction, adjuvant CT	L2	13 mo	Low back pain, weakness	RT	Walk independently	Base of skull	Few wk	Disseminated disease
Vahtsevanos, 2007 [33]	15	80	Lip SCC, G2	T3 N0 M0	Wide excision, rim resection, reconstruction	T6-T7, T12-L1	9 mo	Intense back pain	RT	-	Auxillary LN	13 mo	-
	16	39	Lip SCC, G2	T2 N0 M0	Wide excision, reconstruction, adjuvant RT (44 Gy)	T10	20 mo	-	CT (cisplatin, 5FU)	No response	Multiple foci	2 mo	-
	17	39	Lip SCC, G3	T2 Nx Mx	Wide excision, reconstruction	L4-L5	21 mo	-	RT, CT (paclitaxel, carboplatin)	-	Multiple foci	7 mo	-
Törnwall, 2008 [19]	18	44	Tongue SCC	T1 N0 M0	Partial glossectomy	T11-	7 yr	paraparesis	RT	-	Skull base, neck	Few wk	Sepsis
Yu, 2008 [35]	19	49	Tongue SCC	T4a N2b M0	Left radical neck dissection, suprahyoid neck dissection, hemiglossectomy, reconstruction	C5-C6	9 mo	Numbness, weakness, pain	Steroid, RT	No response – aggravated intolerable pain	Cervical LN, skull base, lung	6 wk	-

“SCC”: Squamous cell carcinoma. “RT”: Radiation therapy. “CT”: Chemotherapy. “CR”: Chemoradiation.” Grade”: Greenberg Grade. “Gy”: Gray. “yr”: year(s). “mo”: month(s). “wk”: week(s). “d”: day(s). “-“: Not given.

**Table 3:** Patients with acinic cell carcinoma as primary head and neck cancer

**Author, year**	**Patient no.**	**Patient age**	**Primary site, grade**	**Primary stage**	**Intervention for primary site**	**Location of spinal metastasis**	**Time frame of spinal metastasis**	**Signs + symptoms of spinal metastasis**	**Intervention for spinal metastasis**	**Outcome**	**Other metastasis**	**Mortality**	**Cause of death**
Vidyadhara, 2007 [24]	1	40	Parotid acinic cell carcinoma	-	Excision	T4	4 mo	Back pain and girdle pain	Decompression, CT (cisplatin, 5FU, epirubicin), RT	Improved, developed lagophthalmos at 6 mo	Sphenoid bone	-	-
Le Manarc’h, 2009	2	65	Parotid acinic cell carcinoma	T1 N0 M0	Complete resection, RT (50 Gy)	L1, L5	6 yr	Low back pain, with radiation to left leg	Arteriography + embolisation, excision, adjuvant RT	No recurrence at 6 mo	None	-	-

“RT”: Radiation therapy. “CT”: Chemotherapy. “CR”: Chemoradiation.” Grade”: Greenberg Grade. “Gy”: Gray. “yr”: year(s). “mo”: month(s). “wk”: week(s). “d”: day(s). “-“: Not given.

**Table 4:** Patients with adenoid cystic carcinoma (ACC) as primary head and neck cancer

**Author, year**	**Patient no.**	**Patient age**	**Primary site, grade**	**Primary stage**	**Intervention for primary site**	**Location of spinal metastasis**	**Time frame of spinal metastasis**	**Signs + symptoms of spinal metastasis**	**Intervention for spinal metastasis**	**Outcome**	**Other metastases**	**Mortality**	**Cause of death**
Riela, 1983 [15]	1	54	Submandibular ACC	-	Operation, RT	T12-L1	17 yr	Back pain, weakness, numbness	Laminectomy, complete resection	Improved symptoms at 6 mo	-	-	-
Preciado, 2002 [20]	2	31	Tongue base ACC	T3 N2b	-	T10-L2	52 mo	Low back pain	CT (etoposide), RT (36 Gy), steroids	Continued pain, worsening; improved pain	Lung	5 mo	-
Birkeland, 2003 [14]	3	55	Submandibular gland ACC	-	Resection, adjuvant RT	L3-L4	7 yr	Low back pain with radiation to left groin	Decompression, RT	Paresis, numbness at all extremities	Cranial, skin	-	-

“ACC”: Adenoid cystic carcinoma. “RT”: Radiation therapy. “CT”: Chemotherapy. “CR”: Chemoradiation.” Grade”: Greenberg Grade. “Gy”: Gray. “yr”: year(s). “mo”: month(s). “wk”: week(s). “d”: day(s). “-“: Not given.

**Table 5:** Patients with carcinoma ex pleomorphic adenoma as primary head and neck cancer

**Author, year**	**Patient no.**	**Patient age**	**Primary site, grade**	**Primary stage**	**Intervention for primary site**	**Location of spinal metastasis**	**Time frame of spinal metastasis**	**Signs + symptoms of spinal metastasis**	**Intervention for spinal metastasis**	**Outcome**	**Other metastasis**	**Mortality**	**Cause of death**
Thomas, 1965 [30]	1	74	Parotid carcinoma ex pleomorphic adenoma	-	Excision, removal of recurrences	T7	8 yr	Weakness, back pain, increased micturition	Laminectomy	No response	None	9 d	GI haemorrhage, benign gastric ulcer
	2	63	Parotid carcinoma ex pleomorphic adenoma	-	Excision, adjuvant RT, removal of recurrences	T8	26 yr	Back pain, complete paralysis	Laminectomy	Paralysis persisted, died soon after op	-	No time given	-
Manoj-Thomas, 2006 [13]	3	58	Parotid carcinoma ex pleomorphic adenoma	-	Superficial parotidectomy	L4	7 yr	Low back pain with radiation to left leg	RT	Improved pain. Hypoaesthesia at 18 mo	Left acetabulum, Lungs	-	-
Ye, 2007 [12]	4	28	Submandibular carcinoma ex pleomorphic adenoma	-	-	T10, L3	40 yr	Leg weakness	Excision, RT (25 Gy)	Improved, no recurrence on follow-up MRI	Forearm skin, scalp, frontal bone, occipital bone, lungs	-	-

“RT”: Radiation therapy. “CT”: Chemotherapy. “CR”: Chemoradiation.” Grade”: Greenberg Grade. “Gy”: Gray. “yr”: year(s). “mo”: month(s). “wk”: week(s). “d”: day(s). “-“: Not given.

**Figure 1:**
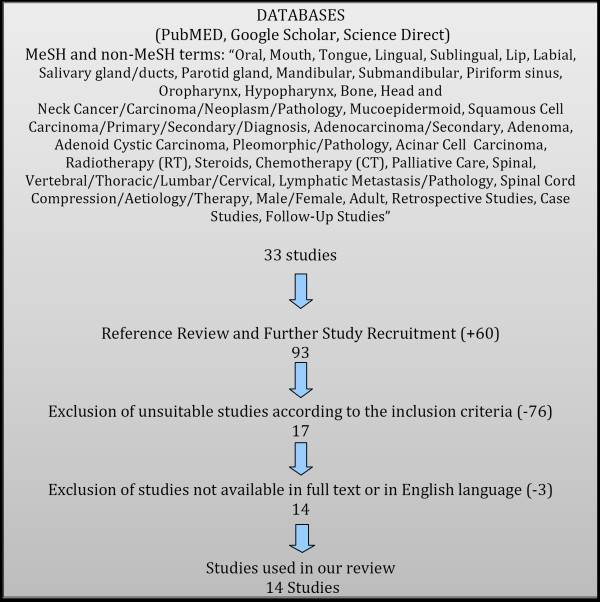
Research criteria.

The search protocol resulted in fourteen articles finally selected for inclusion in this systematic review; consisting of twelve case reports/studies and two retrospective cohort studies all discussing head and neck cancers with spinal metastasis (Figure1). In total, the studies included 28 patients. Due to the rarity of the condition and dearth of publications, this study was unable to limit the year that articles could be produced and therefore has a broad range of publications ranging from one produced in 1965 to more recent ones in 2009. Two independent reviewers ensured that appropriate articles were selected that met the above criteria.

## Results

Our search protocol retrieved 14 articles published from 1965 to 2009 (Table1, 2, 3, 4, and 5). Reported cases of spinal metastasis with primary head and neck cancers were collected, identifying 28 patients in total. Two studies were retrospective cohort studies, and 12 were case studies/reports. Studies varied in terms of location, patient ages and cancer types. Nineteen patients had squamous cell carcinoma (SCC) (Table 2), two had acinic cell carcinoma (Table 3), three had adenoid cyctic carcinoma (ACC) (Table 4), and four had carcinoma ex pleomorphic adenoma (Table 5). Age (at the time of diagnosis) varied from 28 to 80 (Table2, 3, 4 and 5).

In terms of primary site management, surgical excision was performed in fifteen patients, with five patients receiving adjuvant RT, and one patient receiving adjuvant CT. Two patients received RT and CT, one patient received CT as well as chemoradiotherapy (CR), and three patients received RT only. RT was administered to a total of ten patients, and CT was given to four patients in total. Primary site management were not reported in six patients. Cisplatin and 5-fluorouracil (5-FU) were used as the chemotherapeutic agents (Table2, 3, 4 and 5).

Spinal metastasis were most commonly seen in the thoracic region, with seventeen patients. Cervical spine lesions were seen in eight patients, and lumbar spine lesions were seen in twelve patients. Most commonly reported spinal symptoms were back pain and weakness, with sixteen patients reporting each of the symptoms. Three patients experienced numbness. Spinal symptoms were not stated in three patients (Table2, 3, 4 and 5).

Excision of the spinal metastasis was performed in three patients, and two of the three patients received adjuvant radiotherapy. Decompression was performed in seven patients, and four patients were given RT and CT. Twenty-two patients in total received radiotherapy, five received chemotherapy, and seven received steroids. In terms of chemotherapy, cisplatin and 5-FU were used in conjunction in four patients, and one patient received epirubicin on top of the two agents. Etoposide was administered in one patient, and another patient received paclitaxel and carboplatin (Table2, 3, 4 and 5).

Fifteen patients reported improved symptoms after intervention, and ten reported no response or worsened symptoms. Outcomes of intervention were not given in three patients. Within the ten patients with no response or worsened symptoms, two patients received decompression laminectomy only, and another patient received decompression and RT. RT, CT and steroids were given to one patient, and another patient received RT and steroids. Two patients received RT only, and one received CT only. One patient refused surgical procedures and RT, therefore received steroids only (Table2, 3, 4 and 5).

Seventeen patients were found to have other metastasis. Eight patients had metastasis to the skull, and another patient to the acetabulum. Lung metastasis was seen in six patients, regional metastasis including lymph nodes were seen in five patients, and two patients had metastasis in multiple foci. Five patients had no metastasis other than to the spine, and were not reported in six patients (Table2, 3, 4 and 5).

Mortality rate was 89.5% in patients with head and neck SCC as primary cancer, with variable follow-up periods (few months to few years). The mean time from treatment of spinal metastasis to mortality was 3.4 months in reported cases. Patient 14 from Lee et al. [32], and patient 18 from Tornwall et al. [19], are not included in the mean value, as the authors have failed to state numerative figures. Mean time from primary cancer to spinal metastasis was 15.0 months. Patient 2 and 4 from Ampil et al. [31], have not been included because these values are not stated. Cause of death is not given in the majority, but three have died from disseminated disease, and one from sepsis (Table 2).

Within SCC group, two patients from Ampil et al. [31], (2 and 4) were alive at different follow-up periods (5 months and 88 months), and both had “metachronous” presentation of primary cancer and spinal metastasis, but the exact time frame is not given. The other two patients from Ampil et al. [31], (1 and 3) had “synchronous” presentation of the primary and the metastasis, and both have died at 4 months and 1 month, respectively (Table 2).

There were only three other mortalities in non-SCC cancers. These were patient 2 in Preciado et al. [20], and patients 1 and 2 in Thomas et al. [30]. However, patient 1 in Thomas et al. [30], died of gastrointestinal haemorrhage from benign gastric ulcer, unrelated to the cancer (Table4 and 5). The causes of death in the other two are not given. There were no mortalities seen in acinic cell carcinoma. Mean time from primary cancer to metastasis was 9.4 years in ACC, 38 months in acinic cell carcinoma, and 20.3 years in pleomorphic adenoma (Table3,4 and 5).

## Discussion

Our review identified only 12 case studies and 2 small retrospective cohort studies without controls thus any evidence for best practice specific to management spinal metastasis of head and neck cancer is limited (Table1, 2, 3, 4 and 5).

**Figure 2:**
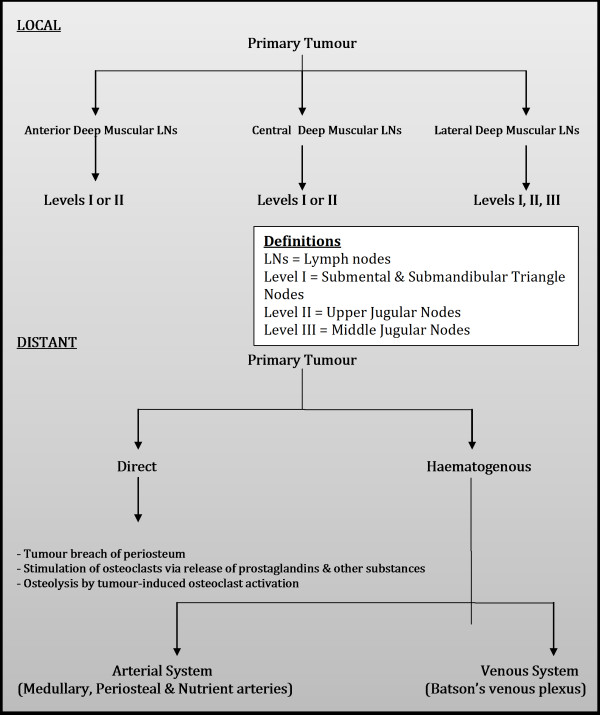
**Pathway of spread of head and neck cancer to bone – squamous cell carcinoma local and distant metastasis to bone.** Adapted from Sano & Myers (2007) [42], Carter & Pittman (1980) [43], Yu et al. (2008) [35] & Yin and Pollock Claire (2005) [44].

**Figure 3:**
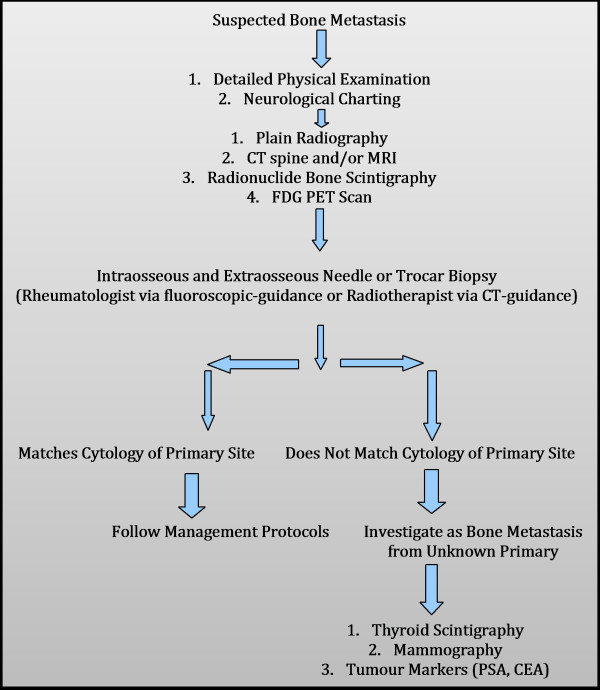
**Pathway of Investigations for bone metastasis from the oral cavity, oropharynx, laryngopharynx and hypopharynx.** Adapted from Destombe et al. (2005) & Yu et al. (2008) [35].

Spinal metastasis from head and neck cancer, although rare, is typically terminal and can significantly impede end-stage quality of life leading to worsening intractable pain, numbness, deformity, and paralysis [1]. Management should aim to improve end-stage quality of life and maintain neurological function [35]. Historically, debate over management of spinal metastasis has considered whether RT, surgery or a combination of the two should form a mainstay of treatment [1-3]; as well as which surgical approach is appropriate in different circumstances. Currently, RT is considered the principle treatment of spinal metastasis and the vast majority of cases we identified in the literature from the last ten years used RT +/− medical adjuvants alone. Surgery has still remained part of treatment in certain cases but any decision to perform surgery must also take into consideration a multitude of factors including age, prognosis, comorbidity, type of tumour, tumour location, operability, spinal stability and risks of intervention amongst others [20,35,37]. Pathway of spread of head and neck cancer to bone have been highlighted in Figure2 (Adapted from Sano & Myers [42], Carter & Pittman [43], Yu et al. [35] and Yin & Pollock Claire [44]. Pathway of investigations for bone metastasis from the head and neck cancers are highlighted in Figure3 (Adapted from Destombe et al. [45] & Yu et al.[35].

### Radiotherapy

External-beam radiotherapy (EBRT) up to a dose of 8 gray (Gy) is known to be effective on patients with pain and neurological deficit secondary to spinal metastasis from a variety of primary tumours [36]. EBRT uses unfocused, wide beam radiation that can cause damage to surrounding structures. This limits the dose of radiation that can be used as there is risk of radiation damage to the spinal cord itself [1,18]. Initial treatment for the head and neck primary may also have already used radiation doses close to tolerance levels [18]. Stereotactic radiotherapy or radiosurgery has been shown to be more effective in reducing pain and neurological deficit as they allow more focused radiation and doses well above 8 Gy without damage to the spinal cord [36,37]. Studies have also found that the effectiveness of EBRT and radiosurgery varies depending on radioresistance of the metastasis so further research comparing effectiveness on different histological types of head and neck cancer would be very welcomed [36].

Systemic radioisotopes with an inclination for osteoclastic bone may also form part of treatment. Strontium-89 or rhenium-189 are two examples of isotopes used with analgesic and antitumour effect. Risk of bone marrow suppression means systemic radioisotope administration is recommended only in those with good bone marrow function and multiple-site spinal metastasis [36]. Only one of our patients – Patient 3 in Preciado [20] - received systemic radioisotope administration, despite several other patients having multi-site metastasis.

### Surgery

Surgery is generally indicated for spinal metastasis in the case of new-onset or progressive neurological deficit secondary to metastatic compression of the spinal cord, spinal instability or collapse by bone destruction, solitary easily resectable spinal metastasis, fracture-dislocation of spine, an enlarging radioresistant tumour, intractable pain unresponsive to nonsurgical intervention (i.e. RT, CT) and a life expectancy of more than 3 months [1,2,13,36,37,40,41]. Preciado [20] has suggested that surgery be performed in case of patients with head and neck neoplasms with unstable spines, no improvement after 2 days of radiotherapy and a life expectancy of greater than 6 months. However, Mendes [18] has questioned the applicability of this approach in all cases. Cervical stability is better maintained by a ventral approach in decompressive surgery, whereas dorsal approaches are more suitable for thoracic and lumbar metastasis [36,37]. Adjuvant radiotherapy has been shown to improve the efficacy of surgery [36]. The surgical options to be considered in spinal metastasis are highlighted in Table 6 (adapted from Bartels [36] and Delank [37]).

**Table 6:** The surgical options to be considered in spinal metastasis

**Presentation**	**Intervention**	**Aims and comments**
Metastasis from highly vascularized primary tumour	Preoperative embolisation of metastasis	- Reduce blood loss in surgery
		- More precise and extensive tumour resection
Dorsal thoracic or lumbar metastasis	Dorsal spine decompression	- Pain relief, neurological improvement
		- Reduce tumour volume
		- Resect structures bordering spinal canal dorsally (laminectomy and hemi-facetectomy)
		- Prevent spinal cord transection
		- Spine stabilisation
Cervical metastasis	Ventral decompression with coroporectomy, vertebral body replacement, and ventral stable-angle plate osteosynthesis	- As for thoracic and lumbar metastasis
Solitary spinal metastasis	Ventral tumour resection	- Removal of malignancy
		- Prognosis good
Vertebral metastasis without neurologically compromise	Vertebroplasty/kyphoplasty	- Stabilisation
		- Pain relief
		- Prevent destruction of vertebral body
		- Possible benefit to neurological function

### Medical therapy

Medical options include analgesics, intravenous steroids, bisphosphonates and chemotherapeutics [20,35-37]. Opioids are especially effective for nociceptive pain; gabapentin, Amitriptyline and doxepin are useful in cases of neuropathic type pain [36]. IV steroids also relieve pain and improve neurological symptoms by reducing vasogenic oedema in the spinal cord contributing to compression [35].

Bisphosphonates reduce metastatic bone complications such as fracture and pain and moderate hypercalcemia by reducing the action of osteoclasts [35-37]. Only one of the patients (Vahtsevanos et al. [33] patient 3) in the studies we have reviewed was treated with bisphosphonates. This may indicate an area to be explored in the future with regards to possible benefits for head and neck cancer spinal metastasis.

Chemotherapeutic agents have been used with some patients in our series as an adjuvant to radiotherapy or surgery [20,33,34]. In the one case where they were used alone as primary treatment, no response was seen [33]. The role of chemotherapy in spinal metastasis management of head and neck cancer needs to be explored further [20].

As spinal metastasis from head and neck cancer is very rare there is very little, if any, high quality evidence for best practice. Our extensive literature search only produced 14 papers from the last 47 years, 12 of which were case studies/reports. We were therefore forced to rely on literature relating to treatment recommendations for spinal metastasis from other primary cancers to assess the range of treatments in use [36,37]. In recent years, there have been more case studies/reports discussing the treatment of head and neck cancer spinal metastasis [13,18,20,31,35]. These case studies/reports, in general, have not used a systematic approach to improve assessment of treatment outcomes. Such a systematic approach might be use of a standardized neurological scoring system to assess functional improvements from interventions or a standardised assessment of pain. Histology of head and neck cancers is diverse: cancers of the buccal mucosa are overwhelmingly SCC, whilst salivary gland cancers present with a more mixed pattern. This makes generalisations about the best approach for management spinal metastasis from the head and neck problematic. The inclusion of other head and neck cancers (i.e. nasopharynx, thyroid, cranial sinuses, brain and eyes cancers) may even complicates the problem.

All patients with suspected bone metastasis must be investigated thoroughly. There should be a high-index of suspicion in any patient with a previous history of cancer presenting with back pain [15]. Investigation should begin with a thorough examination and systematic assessment of neurological function. Imaging should include plain radiography, CT of spine and/or MRI, radionuclide bone scintigraphy and FDG PET scan. An intraosseous and extraosseous needle or trocar biopsy may then be performed to determine if metastasis matches cytology of suspected primary site.

**Figure 4:**
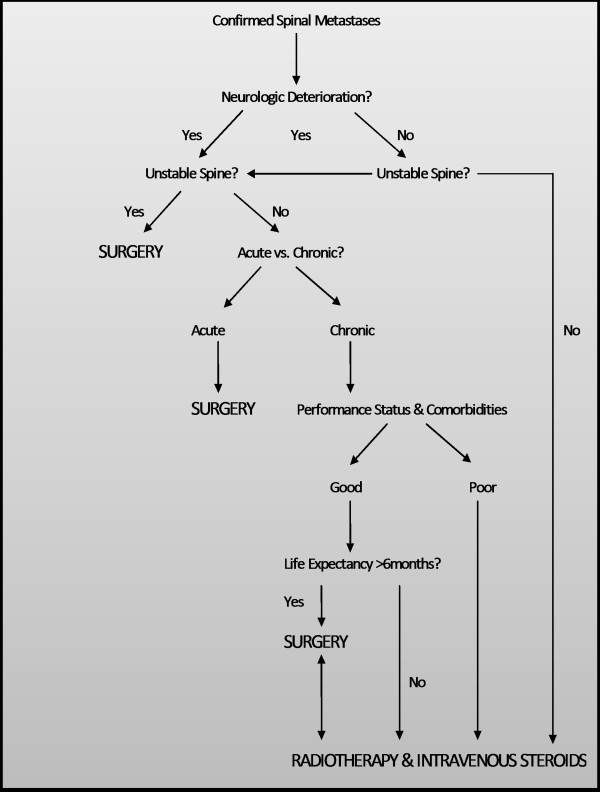
**Management pathway of spinal metastasis.** Adapted from Preciado et al. (2002) [20].

There is inadequate evidence for a treatment protocol to be recommended. Our recommendations are based on current evidence for spinal metastasis from all primary sites including head and neck and non-head and neck. Radiotherapy should be used in all patients with non-radioresistant tumours and sufficient health and prognosis to warrant radiotherapy [36,37]. Surgery should be considered in all patient cases of new-onset or progressive neurological deficit secondary to metastatic compression of the spinal cord, spinal instability or collapse by bone destruction, solitary easily resectable spinal metastasis, fracture-dislocation of spine, an enlarging radioresistant tumour, intractable pain unresponsive to nonsurgical intervention and a life expectancy of more than 3 months [13,36,37,40,41]. Effectiveness of bisphophonates has not been explored in this context and needs to be investigated further. Management pathway of spinal metastasis is highlighted in Figure4 (Adapted from Preciado et al. [20]).

## Conclusions

It is difficult to draw a decisive conclusion for best practice in treatment of spinal metastasis for head and neck cancers. Firstly, head and neck cancers are of diverse histology and vary widely in aggressiveness. Secondly, there is no good quality evidence for which interventions are most effective. More research is needed with controls and systematic assessment of outcomes in order to determine the best mix of medical therapy, radiotherapy and surgery for different patient groups. This will remain difficult given the rarity of spinal metastasis from head and neck cancers.

## Competing interests

The authors declare that they have no competing interests.

## Authors’ contributions

GMT, HC, MAU, SS, AK, WJ, PG contributed to conception and design, carried out the literature research, manuscript preparation and manuscript review. All authors read and approved the final manuscript.

## References

[B1] Jerjes W Upile T Petrie A Riskalla A Hamdoon Z Vourvachis M Karavidas K Jay A Sandison A Thomas GJ Kalavrezos N Hopper C Clinicopathological parameters, recurrence, locoregional and distant metastasis in 115 T1-T2 oral squamous cell carcinoma patients Head Neck Oncol 2010 2 9 10.1186/1758-3284-2-9 20406474 PMC2882907

[B2] Saman DM A review of the epidemiology of oral and pharyngeal carcinoma: update Head Neck Oncol 2012 4 1 10.1186/1758-3284-4-1 22244087 PMC3292826

[B3] Upile T Jerjes W Al-Khawalde M Radhi H Sudhoff H Oral sex, cancer and death: sexually transmitted cancers Head Neck Oncol 2012 4 1 31 10.1186/1758-3284-4-31 22673108 PMC3448502

[B4] Lagha A Chraiet N Ayadi M Krimi S Allani B Rifi H Raies H Mezlini A Systemic therapy in the management of metastatic or advanced salivary gland cancers Head Neck Oncol 2012 4 1 19 10.1186/1758-3284-4-19 22558945 PMC3414773

[B5] Rodriguez T Altieri A Chatenoud L Gallus S Bosetti C Negri E Franceschi S Levi F Talamini R Vecchia CL Risk factors for oral and pharyngeal cancer in young adults Oral Oncol 2004 40 207 213 10.1016/j.oraloncology.2003.08.014 14693246

[B6] Llewellyn CD Linklater K Bell J Johnson NW Warnakulasuriya S An analysis of risk factors for oral cancer in young people: a case–control study Oral Oncol 2004 40 304 313 10.1016/j.oraloncology.2003.08.015 14747062

[B7] Jan JC Hsu WH Liu SA Wong YK Poon CK Jiang RS Jan JS Chen IF Prognostic factors in patients with Buccal Squamous Cell Carcinoma: 10 year experience J Oral Maxillofac Surg 2011 69 396 404 10.1016/j.joms.2010.05.017 21238843

[B8] Shah JP Gil Z Current concepts in management of oral cancer – surgery Oral Oncol 2009 45 394 401 10.1016/j.oraloncology.2008.05.017 18674952 PMC4130348

[B9] Vaidya AM Petruzzelli GJ Clark J Emami B Patterns of spread in recurrent head and neck squamous cell carcinoma Otolaryngol Head Neck Surg 2001 125 4 393 396 10.1067/mhn.2001.117715 11593178

[B10] Funk GF Karnell LH Robinson RA Zhen WK Trask DK Hoffman HT Presentation, treatment, and outcome of oral cavity cancer: a National Cancer Data Base report Head Neck 2002 24 2 165 180 10.1002/hed.10004 11891947

[B11] Lee DH Kim MJ Roh JL Kim SB Choi SH Nam SY Kim SY Distant Metastases and Survival Prediction in Head and Neck Squamous Cell Carcinoma Otolaryngol Head Neck Surg 2012 147 1 6 10.1177/0194599812446282 22581637

[B12] Ye HH Cho CW Jeon MY Kim DJ Craniospinal Metastasis from a Metastasizing Mixed Tumor of Salivary Gland: Unusual Presentation J 2007 41 186 189 10.3340/jkns.2007.41.3.186

[B13] Manoj-Thomas A Dabke H Hammer K Attanoos R Ahuja S Spinal metastasis from a primary parotid carcinoma: a case report Joint Bone Spine 2006 73 5 573 575 10.1016/j.jbspin.2005.12.010 16952477

[B14] Birkeland S Spinal Metastasis of Submandibular Gland Adenoid Cystic Carcinoma: A Case Report Surg Neurol 2003 60 265 266 10.1016/S0090-3019(03)00294-5 12922052

[B15] Riela AR Meyer D McCool JA Pikula L Metastatic-adenoid cystic carcinoma of the major salivary glands presenting as a spinal cord tumor Surg Neurol 1983 19 4 365 368 10.1016/0090-3019(83)90246-X 6301086

[B16] Le Manac'h AP Rousselet MC Massin P Audran M Levasseur R Extraspinal sciatica revealing late metastatic disease from parotid carcinoma Joint Bone Spine 2010 77 1 64 66 10.1016/j.jbspin.2009.11.012 20022535

[B17] Warnakulasuriya S Review: Global epidemiology of oral and oropharyngeal cancer Oral Oncol 2009 45 309 316 10.1016/j.oraloncology.2008.06.002 18804401

[B18] Mendes L Nutting M Harrington J Residual or recurrent head and neck cancer presenting with nerve root compression affecting the upper limbs Br J Radiol 2004 77 688 690 10.1259/bjr/16836733 15326051

[B19] Törnwall J Snäll J Mesimäki K A rare case of spinal cord metastases from oral SCC Br J Oral Maxillofac Surg 2008 46 594 595 10.1016/j.bjoms.2008.02.004 18359540

[B20] Preciado DA Sebring LA Adams GL Treatment of Patients With Spinal Metastases From Head and Neck Neoplasms Arch Otolaryngol Head Neck Surg 2002 128 539 543 10.1001/archotol.128.5.539 12003584

[B21] Harrington KD Metastatic disease of the spine J Bone Joint Surg Am 1986 68-A 1110 1115 3745256

[B22] Eastley N Newey M Ashford RU published online ahead of printMay 1 2012] The role of the orthopaedic and spinal surgeon Surg Oncol 2012 http://www.sciencedirect.com/science/article/pii/S0960740412000242Accessed May 20, 2012 10.1016/j.suronc.2012.04.001 22554913

[B23] Perrin RG Laxton AW Metastatic spine disease: epidemiology, pathophysiology, and evaluation of patients Neurosurg Clin N Am 2004 15 4 365 373 10.1016/j.nec.2004.04.018 15450871

[B24] Harel R Angelov L Spine metastases: current treatments and future directions Eur J Cancer 2010 46 15 2696 2707 10.1016/j.ejca.2010.04.025 20627705

[B25] Molina CA Gokaslan ZL Sciubba DM Diagnosis and management of metastatic cervical spine tumors Orthop Clin North Am 2012 43 1 75 87 10.1016/j.ocl.2011.08.004 22082631

[B26] Constans JP Dde Divitiis E Donzelli R Spinal metastases with neurological manifestations. Review of 600 cases J Neurosurg 1983 59 1 111 118 10.3171/jns.1983.59.1.0111 6864265

[B27] Gilbert RW Kim JH Posner JB Epidural spinal cord compression from metastatic tumor: diagnosis and treatment Ann Neurol 1978 3 1 40 51 10.1002/ana.410030107 655653

[B28] Sciubba DM Petteys RJ Dekutoski MB Fisher CG Fehlings MG Ondra SL Rhines LD Gokaslan ZL Diagnosis and management of metastatic spine disease J Neurosurg Spine 2010 13 1 94 108 10.3171/2010.3.SPINE09202 20594024

[B29] Costachescu B Popescu CE Modern management in vertebral metastasis Romanian Neurosurgery 2010 27 4 432 437

[B30] Thomas WH Coppola ED Distant Metastases from mixed tumors of the salivary glands Am J Surg 1965 109 724 730 10.1016/S0002-9610(65)80042-3 14283329

[B31] Ampil FL Nanda A Aarstad RF Hoasjoe DK Chin HW Hardjasudarma M Spinal epidural compression in head and neck cancer. Report of five cases J Craniomaxillofac Surg 1994 22 1 49 52 10.1016/S1010-5182(05)80296-3 8175998

[B32] Lee KH Halfpenny W Thiruchelvam JK Spinal cord compression in patients with oral squamous cell carcinoma Oral Surg Oral Med Oral Pathol Oral Radiol Endod 2007 103 4 e16 e18 10.1016/j.tripleo.2006.11.041 17275365

[B33] Vahtsevanos K Ntomouchtsis A Andreadis C Patrikidou A Karakinaris G Mangoudi D Papanastasiou G Antoniades K Distant bone metastases from carcinoma of the lip: a report of four cases Int J Oral Maxillofac Surg 2007 36 2 180 185 10.1016/j.ijom.2006.07.010 17223312

[B34] Vidyadhara S Shetty AP Rajasekaran S Widespread metastases from acinic cell carcinoma of parotid gland Singapore Med J 2007 48 1 e13 e15 17245497

[B35] Yu EH Wu CH Lo WL Kao SY Chang CS Cervical vertebrae metastases in oral squamous cell carcinoma: a case report Chin 2008 19 65 70

[B36] Bartels RH van der Linden YM van der Graaf WT Spinal extradural metastasis: review of current treatment options CA Cancer J Clin 2008 58 4 245 259 10.3322/CA.2007.0016 18354080

[B37] Delank KS Wendtner C Eich HT Eysel P The treatment of spinal metastases Dtsch Arztebl Int 2011 108 5 71 79 quiz 80 21311714 10.3238/arztebl.2011.0071 PMC3036978

[B38] Singh K Samartzis D Vaccaro AR Andersson GB An HS Heller JG Current concepts in the management of metastatic spinal disease. The role of minimally-invasive approaches J Bone Joint Surg Br 2006 88 4 434 442 10.1302/0301-620X.88B4.17282 16567775

[B39] Finn MA Vrionis FD Schmidt MH Spinal radiosurgery for metastatic disease of the spine Cancer Control 2007 14 4 405 411 17914341 10.1177/107327480701400411

[B40] Ryken TC Eichholz KM Gerszten PC Welch WC Gokaslan ZL Resnick DK Evidence-based review of the surgical management of vertebral column metastatic disease Neurosurg Focus 2003 15 5 E11 15323468 10.3171/foc.2003.15.5.11

[B41] Tonini G Vincenzi B Spoto C Santini D Complications in surgical management of cervical spinal metastases Pitfalls in Cervical Spine Surgery 2010 Section I 29 43

[B42] Sano D Myers JN Metastasis of squamous cell carcinoma of the oral tongue Cancer Metastasis Rev 2007 26 3–4 645 662 17768600 10.1007/s10555-007-9082-y

[B43] Carter RL Pitman NR Squamous carcinomas of the head and neck: some patterns of spread J R Soc Med 1980 73 420 427 7230216 10.1177/014107688007300606 PMC1437597

[B44] Yin JJ Pollock Claire B Mechanisms of cancer metastasis to the bone Cell Res 2005 15 1 57 62 10.1038/sj.cr.7290266 15686629

[B45] Destombe C Botton E Le Gal G Roudaut A Jousse-Joulin S Devauchelle-Pensec V Saraux A Investigations for bone metastasis from an unknown primary Joint Bone Spine 2007 74 1 85 89 10.1016/j.jbspin.2006.05.009 17218141

